# Rainfall frequency and uncertainty analysis in arid regions using machine learning and statistical distribution models based on a 91-year rainfall record

**DOI:** 10.1038/s41598-026-68681-6

**Published:** 2026-09-15

**Authors:** Mohammed Hagage

**Affiliations:** Engineering Applications and Water Division, National Authority for Remote Sensing and Space Sciences (NARSS), El-Nozha El-Gedida, P.O. Box 1564, Cairo, Egypt

**Keywords:** Annual maximum daily rainfall (AMDR), Arid regions, Bootstrap uncertainty analysis, Generalized extreme value (GEV), Uncertainty quantification, Rainfall return periods, Random forest, XGBoost regressor, Climate sciences, Environmental sciences, Hydrology, Mathematics and computing, Natural hazards

## Abstract

Accurate estimation of rainfall depths associated with standard return periods is fundamental for hydraulic design, flood risk assessment, and water resources management, particularly in arid regions. Statistical extreme value distributions have long been the standard approach for rainfall frequency analysis, whereas machine learning (ML) has recently emerged as a potential alternative. This study presents a comprehensive comparison between twenty-five statistical distribution models and three supervised ML regressors—Support Vector Machine (SVM), Random Forest (RF), and Extreme Gradient Boosting Regressor (XGBR)—for Annual Maximum Daily Rainfall (AMDR) frequency analysis using a 91-year record (1931–2021) from the Suez meteorological station, Egypt. Prior to frequency analysis, the AMDR series was verified to satisfy the assumptions of stationarity and serial independence using the Mann–Kendall test, Sen's slope estimator, and Lag-1 autocorrelation analysis. Statistical distribution models were implemented in HEC-SSP using Weibull plotting positions, whereas the ML models were developed in Python using exceedance probability as the sole predictor to enable a direct and consistent comparison with statistical frequency models. Model performance was evaluated using the coefficient of determination (R^2^), mean squared error (MSE), root mean squared error (RMSE), and mean absolute error (MAE), while predictive uncertainty was quantified using 90% bootstrap confidence intervals. The results demonstrate that statistical distribution models consistently outperformed the ML regressors across all performance metrics. The Generalized Extreme Value distribution fitted using Maximum Likelihood Estimation (GEVMLE) achieved the highest predictive performance (R^2^ = 0.968; RMSE = 1.512 mm), followed by GEVLM and GLLM. In contrast, the ML models exhibited substantially lower predictive accuracy (R^2^ = 0.280 for RF, 0.258 for XGBR, and 0.087 for SVM) and showed limited capability for extrapolating rainfall extremes beyond the observed data range at longer return periods (T ≥ 25 years). The ML models also produced comparatively narrow confidence intervals, reflecting the limited variability represented within the training domain, whereas the GEV-based statistical models provided more realistic uncertainty estimates for extreme rainfall quantiles. Overall, the findings indicate that extreme value distributions, particularly the GEV models, remain more robust than ML regressors for rainfall frequency analysis when only long-term single-variable rainfall records are available. GEVMLE is therefore recommended for rainfall frequency analysis at the Suez station and similar arid coastal environments. Future research should investigate hybrid frameworks that integrate extreme value theory with machine learning using multi-variable climatic predictors to improve extrapolation capability and uncertainty representation.

**Supplementary Information:**

The online version contains supplementary material available at https://doi.org/10.1038/s41598-026-68681-6.

## Introduction

Rainfall frequency analysis is a fundamental component of hydrological and water resources engineering, providing design rainfall estimates for specified return periods that support the planning and design of hydraulic infrastructure, including flood control structures, urban drainage systems, and dam spillways^1–3^. These estimates are commonly obtained by fitting probability distributions to Annual Maximum Daily Rainfall (AMDR) records and estimating rainfall depths associated with selected return periods. Their accuracy is critical because underestimation may increase flood risk, whereas overestimation can lead to unnecessarily conservative and costly infrastructure^4–6^.

Reliable estimation of extreme rainfall is particularly challenging in arid and semi-arid regions, where rainfall is characterized by low annual totals, high temporal variability, infrequent but intense storm events, and relatively short observational records^7^. The Gulf of Suez, located within Egypt's arid coastal zone, exhibits these characteristics, with sporadic convective storms frequently generating destructive flash floods despite the generally dry climate^1,8^. Such conditions require extrapolation beyond the observed data range, increasing uncertainty and sensitivity to model selection. Consequently, accurate rainfall frequency analysis remains essential for flood-risk mitigation, hydraulic design, and sustainable water resources management in arid environments^6,9,10^.

Extreme rainfall frequency analysis is primarily founded on extreme value theory, which establishes the Generalized Extreme Value (GEV) distribution as the asymptotic model for block maxima^11^. Consequently, GEV and its special cases have become the most widely adopted distributions for hydrological frequency analysis^12^. Nevertheless, several alternative distributions, including Log-Normal, Log-Pearson Type III, Generalized Logistic, Generalized Pareto, Gamma, and Exponential distributions, have also demonstrated satisfactory performance under different hydroclimatic conditions. For example, GEV-based models have frequently provided reliable estimates in arid and semi-arid environments, while Log-Pearson Type III and Generalized Logistic distributions have shown competitive performance depending on local rainfall characteristics^4,6,9,13^. These findings indicate that no single probability distribution is universally optimal, emphasizing the importance of evaluating alternative models for individual study areas.

The reliability of rainfall frequency analysis depends not only on the selected probability distribution but also on the parameter estimation method. Maximum Likelihood Estimation (MLE) and L-Moment Estimation (LME) are among the most widely adopted techniques, with L-moments providing greater robustness for short and highly variable records, whereas MLE generally offers statistically efficient parameter estimates for extreme value models^11,14–16^. Although these statistical approaches have been successfully applied in numerous hydrological studies, their performance depends on selecting an appropriate probability distribution and accurately estimating model parameters. These challenges become more pronounced in arid environments, where highly variable rainfall records and the estimation of rare events through extrapolation can substantially increase predictive uncertainty. Consequently, increasing attention has been directed toward data-driven machine learning techniques as complementary tools for hydrological analysis.

Recent advances in machine learning (ML) have expanded its application across a broad spectrum of hydrological problems, including rainfall prediction, rainfall–runoff modelling, flood forecasting, and coastal flood prediction^17–23^. Unlike conventional statistical methods, ML algorithms can capture complex nonlinear relationships without requiring assumptions regarding the underlying probability distribution, making them attractive for data-driven hydrological modelling.

Despite these advantages, the application of ML to rainfall frequency analysis remains relatively limited. Most existing studies have focused on predictive hydrological modelling rather than estimating design rainfall associated with specified return periods. Furthermore, the performance of supervised ML models depends strongly on the quantity and representativeness of training data, which may restrict their ability to extrapolate beyond the observed data range to estimate rare rainfall extremes. This limitation is particularly important for rainfall frequency analysis based on limited single-variable datasets, where extrapolation is fundamental for estimating design rainfall at long return periods^17,21,24,25^.

In addition to predictive accuracy, uncertainty quantification represents an essential component of rainfall frequency analysis because design rainfall estimates are influenced by sampling variability, parameter estimation, and model selection^6,11^. Bootstrap resampling has therefore become a widely adopted approach for estimating confidence intervals of rainfall quantiles, particularly when long observational records are unavailable or sample sizes are limited^6,10^. However, uncertainty has rarely been evaluated alongside both statistical distribution models and ML regressors within a unified comparative framework.

Despite considerable progress in both statistical frequency analysis and machine learning applications, several important research gaps remain. Previous studies have generally focused either on comparing statistical probability distributions for rainfall or flood frequency analysis (e.g.^6^) or on applying ML techniques to rainfall prediction, rainfall–runoff simulation, and flood forecasting (e.g.^17,18,21^). Consequently, comprehensive evaluations that directly compare statistical distribution models and ML regressors for rainfall frequency analysis under identical analytical conditions remain limited, particularly in arid environments where reliable design rainfall estimation is critical for hydraulic infrastructure and flood-risk management^1,8,26^.

To address these gaps, this study develops a unified evaluation framework that directly compares twenty-five statistical distribution models with three widely used machine learning regressors (Support Vector Machine, Random Forest, and Extreme Gradient Boosting Regressor) using the same AMDR dataset, standardized performance metrics, and bootstrap-based uncertainty analysis. This framework enables a consistent assessment of both predictive accuracy and uncertainty, providing further insights into the applicability and limitations of machine learning for rainfall frequency analysis based on limited single-variable datasets.

The proposed framework is subsequently implemented using the 91-year AMDR record from the Suez meteorological station to evaluate the predictive performance and uncertainty of both statistical distribution models and machine learning regressors under identical analytical conditions.

## Materials and methods

**Fig. 1:**
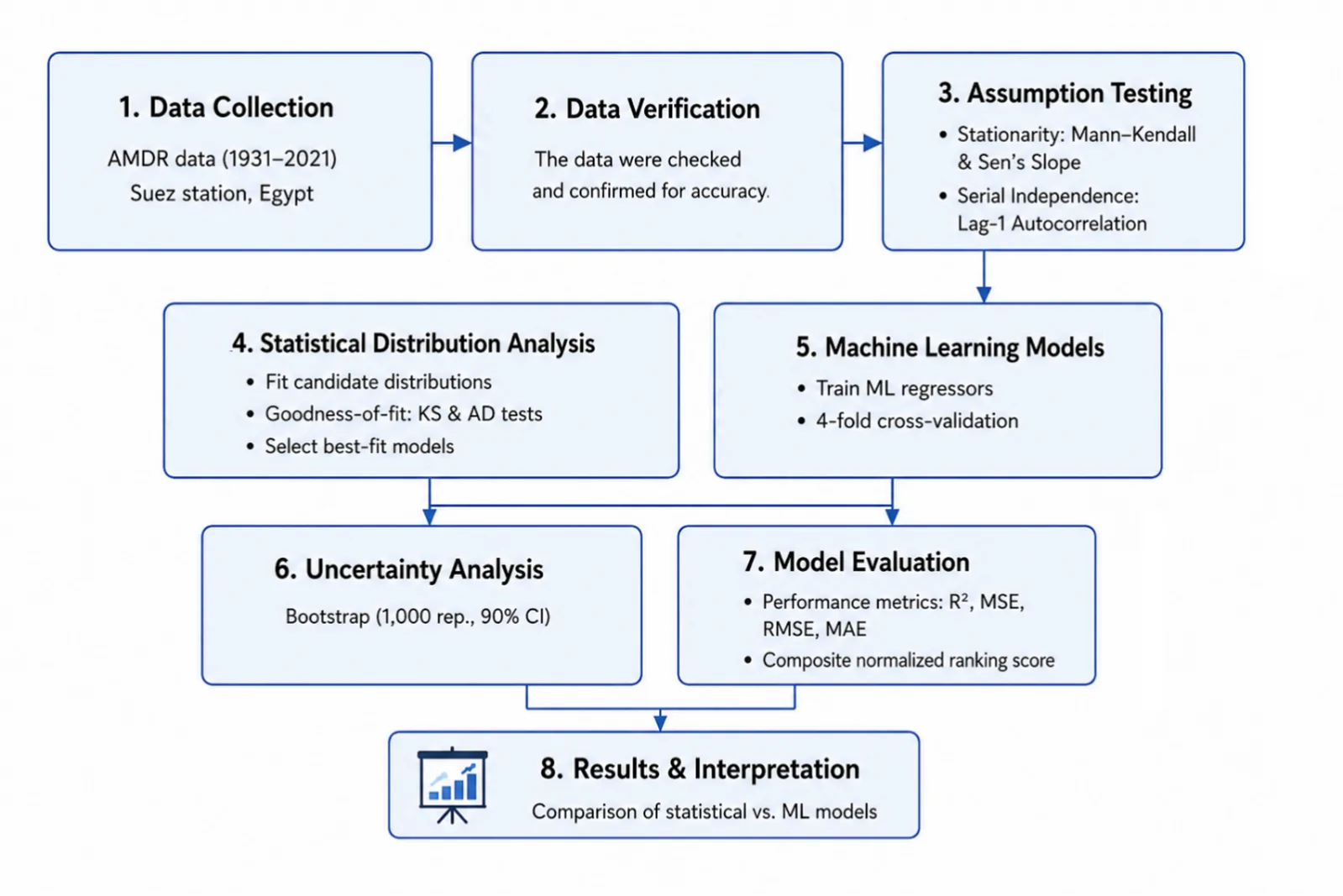
Overview of the methodological framework adopted in this study.

The overall methodological framework adopted in this study is summarized in Fig. 1.

### Meteorological data

**Fig. 2:**
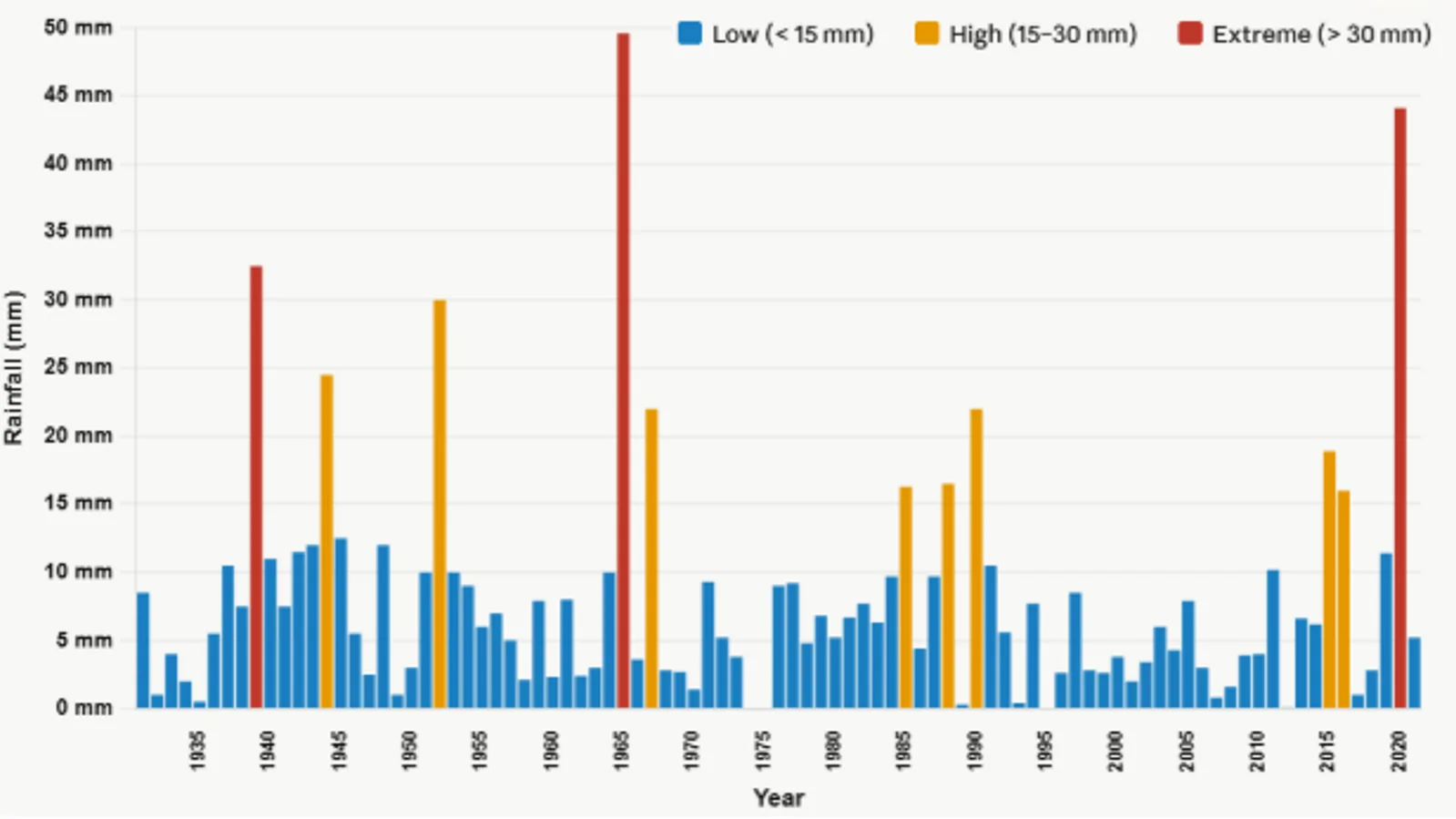
Time series of AMDR (mm) at Suez station (1931–2021).

The Suez meteorological station (29°58′N, 32°33′E; elevation 5 m a.s.l.) is located on the western shore of the Gulf of Suez, at the northern terminus of the Red Sea, within the eastern Egyptian coastal desert. The station occupies a climatologically sensitive transition zone influenced by multiple moisture sources, including the eastern Mediterranean, the Gulf of Suez, and the northern Red Sea. The dataset used in this study consists of the Annual Maximum Daily Rainfall (AMDR) series recorded at Suez station over the period 1931–2021, comprising 91 annual observations. The complete dataset is provided in Table S1 (Supplementary Material), while the temporal variability of the series is illustrated in Fig. 2.

Descriptive statistics of the AMDR series are summarized in Table 1. Skewness and kurtosis were computed using method-of-moments estimators. The series exhibits strong positive skewness (2.61) and high excess kurtosis (10.37), indicating a heavy-tailed distribution characterized by infrequent but high-magnitude rainfall events, consistent with arid-region precipitation regimes. Several extreme rainfall events are evident in the record, including 49.6 mm (1965), 44.1 mm (2020), and 32.5 mm (1939). In contrast, years with zero recorded AMDR (1974, 1975, and 1995) highlight the pronounced inter-annual variability typical of hyper-arid Mediterranean coastal environments. The zero-AMDR years contribute to the lower part of the empirical distribution, whereas the unusually large events, particularly the 1965 and 2020 maxima, exert greater influence on the estimation of the upper tail. Consequently, their inclusion affects the fitted tail behavior of the heavy-tailed distributions and contributes to the differences observed in long-return-period estimates.

**Table 1:** Descriptive statistics of the annual maximum daily rainfall (AMDR) series at Suez station (1931–2021). CV = coefficient of variation.

Parameter	Value	Parameter	Value	Parameter	Value
Record period	1931–2021	Mean (mm)	7.48	Skewness	2.61
Number of years	91	Median (mm)	5.80	Kurtosis	10.37
Maximum (mm)	49.6 (1965)	Std. deviation (mm)	8.39	CV	1.12
Minimum (mm)	0 (1974, 1975, 1995)	25th percentile (mm)	2.60	75th percentile (mm)	10.20

Prior to the rainfall frequency analysis, the AMDR series was evaluated to verify the fundamental assumptions of stationarity and serial independence underlying conventional frequency analysis. Stationarity was assessed using the non-parametric Mann–Kendall trend test together with Sen's slope estimator^27^. The results indicated no statistically significant monotonic trend in the AMDR series (Z =  − 1.1144, p = 0.2651), while Sen's slope (− 0.0228 mm year⁻^1^) indicated only a negligible long-term decrease in rainfall, supporting the applicability of the stationarity assumption. Serial independence was subsequently evaluated using the lag-1 autocorrelation coefficient^28^. The analysis yielded a lag-1 autocorrelation coefficient of 0.0820 with a p-value of 0.4425, and the corresponding 95% confidence interval (− 0.1273 to 0.2842) included zero, indicating the absence of statistically significant serial correlation. These results confirm that the AMDR series satisfies the assumptions of both stationarity and serial independence, supporting the application of conventional rainfall frequency analysis. Potential step changes, regime shifts, or changes in variance were not explicitly evaluated and are acknowledged as additional aspects of stationarity assessment.

### Statistical distribution models

Return periods for all observed AMDR values were computed using the Weibull plotting position formula^29^, which assigns an empirical exceedance probability to the i-th ranked observation in a sample of size n as P = i / (n + 1), where i is the rank in ascending order and n = 91 is the total number of annual observations. The corresponding return period is given by T = 1 / P.

A total of twenty-five statistical distribution models spanning seven parametric families were evaluated using the HEC-SSP (version 2.3.1) framework. Three parameter estimation methods were employed: Maximum Likelihood Estimation (MLE), which maximizes the likelihood function of the observed data given the model parameters^30^, L-moment estimation (LM), which matches theoretical L-moment ratios to their sample counterparts and is known for its robustness to outliers and suitability for limited-length records; and Product Moments (PM), based on conventional moment matching^31^. The evaluated models represent the complete set of probability distributions and parameter estimation methods implemented in HEC-SSP (version 2.3.1) that are commonly used in hydrological frequency analysis^2,32^. Table S2 (Supplementary Material) summarizes the complete HEC-SSP configuration adopted for statistical distribution fitting, goodness-of-fit testing, return period estimation, and bootstrap confidence interval computation.

A dual goodness-of-fit screening procedure was applied to all candidate statistical distributions using the Kolmogorov–Smirnov (KS) and Anderson–Darling (AD) tests^33^. The KS statistics quantify the maximum absolute difference between the empirical and theoretical cumulative distribution functions and is sensitive to discrepancies across the entire distribution. In contrast, the AD statistics assign greater weight to the distribution tails, making it particularly suitable for evaluating extreme rainfall behavior^34,35^. The combined application of both tests therefore provides a rigorous goodness-of-fit assessment by simultaneously evaluating the overall distribution and its upper-tail characteristics.

Model acceptance was assessed using sample-size-dependent critical values corresponding to the present dataset (n = 91). For the KS test, the critical value at the 5% significance level was computed using the asymptotic relationship, ${D}_{\alpha }=\frac{c(\alpha )}{\sqrt{n}}$, where c (0.05) = 1.3581, yielding D_0.05_ = 1.3581/91 = 0.143^36^. For the AD test, the critical value was calculated using the sample-size correction proposed by Yürekli et al.^37^,

1 $$CV=\frac{0.752}{\left(1+0.75/n+2.25/{n}^{2}\right)}$$

which gives CV = 0.746 for n = 91. A statistical distribution was retained for further analysis only when both the KS and AD test statistics were smaller than their corresponding critical values. Based on these criteria, thirteen statistical distribution models satisfied the goodness-of-fit requirements and were subsequently retained for performance evaluation, as presented in Table 2.

**Table 2:** Goodness-of-fit screening results for the thirteen retained statistical distribution models. MLE = maximum likelihood estimation; LM = L-moments; PM = product moments.

No	Distribution models	Estimation methods	Model ID	AD statistic	KS statistic
1	Generalized Pareto	PM	GPPM	0.07	0.08
2	Exponential	PM	EPM	0.3	0.09
3	Log-Logistic	PM	LLPM	0.6	0.07
4	Gamma	PM	GPM	0.09	0.1
5	Gamma	LM	GLM	0.26	0.09
6	Generalized logistic	LM	GLLM	0.3	0.05
7	Generalized extreme value	LM	GEVLM	0.3	0.05
8	Exponential	LM	ELM	0.3	0.09
9	Log-logistic	LM	LLLM	0.6	0.07
10	Generalized extreme value	MLE	GEVMLE	0.3	0.06
11	Exponential	MLE	EMLE	0.3	0.09
12	Shifted exponential	MLE	SEMLE	0.3	0.09
13	Generalized Pareto	MLE	GPMLE	0.4	0.08

### Machine learning models

Three supervised machine learning (ML) regression models were implemented in Python (version 3.14.4) using the scikit-learn (version 1.8.0) and XGBoost (version 3.2.0) libraries: Support Vector Machine (SVM), Random Forest (RF), and Extreme Gradient Boosting Regressor (XGBR). These models represent three widely used ML paradigms that have been extensively applied in hydrological regression problems^23,38,39^. More complex deep learning architectures (e.g., Artificial Neural Networks (ANN) and Long Short-Term Memory (LSTM) networks) were not considered because the available dataset comprised only 91 annual observations, which is insufficient for training high-capacity neural networks without substantially increasing the risk of overfitting^40^. Consequently, the selected models provide a balanced and methodologically appropriate benchmark for comparison with conventional statistical frequency models.

Each model was trained using exceedance probability as the sole predictor variable (X) and Annual Maximum Daily Rainfall (AMDR, mm) as the response variable (y). This simplified input configuration was intentionally adopted as a methodological trade-off to enable a direct, controlled, and methodologically consistent comparison with statistical distribution models, which likewise establish a functional relationship between exceedance probability and rainfall magnitude. Although incorporating additional climatic predictors could potentially improve ML performance, such data were not available at sufficiently high temporal and spatial resolution for the entire 91-year study period. Consequently, the analysis was restricted to the long-term homogeneous rainfall record to ensure methodological consistency and a reliable comparison among models.

Hyperparameter optimization was performed for all three ML models. The final optimized configurations were as follows: the SVM employed a radial basis function (RBF) kernel with C = 100, gamma = “scale”, and epsilon = 0.1, preceded by standardization using StandardScaler. The RF model used 300 trees with no predefined maximum tree depth. The XGBR model was configured with 300 estimators, a learning rate of 0.05, a maximum tree depth of 4, subsample = 0.9, and colsample_bytree = 0.9.

Model generalization performance was evaluated using a 4-fold cross-validation scheme. The 4-fold configuration was selected to provide a balanced compromise between the limited sample size (91 annual observations) and sufficiently large training and testing subsets for reliable model evaluation. The dataset was partitioned into four temporally consecutive folds of approximately equal size, with no random shuffling applied to preserve the chronological structure of the AMDR series. In each iteration, three folds were used for training and one-fold for testing. Performance metrics were computed independently for each fold, and overall model performance was reported as the mean and standard deviation across all folds. Detailed fold-level performance results are provided in Table S3 (Supplementary Material).

### Model performance evaluation

The predictive accuracy of the retained statistical distribution models and the machine learning (ML) regressors was evaluated using the Coefficient of Determination (R^2^), Mean Squared Error (MSE), Root Mean Squared Error (RMSE) and Mean Absolute Error (MAE)^41^. These metrics were used only to provide a common basis for comparing the predictive performance of the two modelling approaches. Statistical distributions were selected independently using the Kolmogorov–Smirnov (KS) and Anderson–Darling (AD) goodness-of-fit tests before applying these performance metrics. These metrics were computed over all observed return periods for the statistical distribution models and across all cross-validation folds for the machine learning (ML) models.

Because the statistical and machine learning approaches follow different estimation and validation frameworks, the same validation procedure was not adopted, and the performance metrics are not intended to reflect identical validation procedures. Thus, the comparison across modelling paradigms should be interpreted as contrasting the fitted performance of the statistical frequency models with the out-of-sample predictive performance of the machine learning models within the adopted experimental framework.

The performance metrics are defined as follows:

2 $$ R^{{2}} \, = \, 1 \, - \, \Sigma \, \left( {y_{i} \, - \, \widehat{y}_{i} } \right) \, ^{{2}} \, / \, \Sigma \, \left( {y_{i} \, - \, \overline{y} } \right) \, ^{{2}} $$

3 $$ MSE \, = \, \left( {1/n} \right) \, \Sigma \, \left( {y_{i} \, - \, \widehat{y}_{i} } \right) \, ^{{2}} $$

4 $$ RMSE \, = \, \surd MSE $$

5 $$ MAE \, = \, \left( {1/n} \right) \, \Sigma \left| {y_{i} \, - \, \widehat{y}_{i} } \right| $$

where yᵢ is the observed AMDR, ŷᵢ is the model-predicted value, ȳ is the observed mean, and n is the number of data points. R^2^ ranges from − ∞ to 1, with values closer to 1 indicating superior explanatory power. MSE, RMSE, and MAE are error-based metrics for which lower values indicate better performance.

### Composite normalized ranking score and sensitivity analysis

To enable objective cross-paradigm model comparison across four metrics with different units and scales, a composite normalized ranking score was computed for each model. The normalization procedure transforms each metric to a common [0, 1] scale and assigns a score of 1 to the best-performing model and 0 to the worst-performing model. The procedure is described in Eqs. 5–9.

Step 1: Normalization of R^2^ (higher is better):

6 $$ R^{2} \_norm \, = \, \left( {R^{2} \, - \, min\left( {R^{2} } \right)} \right) \, / \, \left( {max\left( {R^{2} } \right) \, - \, min\left( {R^{2} } \right)} \right). $$

Step 2: Normalization of error metrics (lower is better; direction is reversed):

7 $$ MSE\_norm \, = \, \left( {max \, \left( {MSE} \right) \, - \, MSE} \right) \, / \, \left( {max \, \left( {MSE} \right) \, - \, min \, \left( {MSE} \right)} \right). $$

8 $$ RMSE\_norm \, = \, \left( {max \, \left( {RMSE} \right) \, - \, RMSE} \right) \, / \, \left( {max \, \left( {RMSE} \right) \, - \, min \, \left( {RMSE} \right)} \right). $$

9 $$ MAE\_norm \, = \, \left( {max \, \left( {MAE} \right) \, - \, MAE} \right) \, / \, \left( {max \, \left( {MAE} \right) \, - \, min \, \left( {MAE} \right)} \right). $$

Step 3: Composite score (equal weighting of all four metrics):

10 $$ Score \, = \, \left( {R^{2} \_norm \, + \, MSE\_norm \, + \, RMSE\_norm \, + \, MAE\_norm} \right) \, / \, 4. $$

This normalization scheme ensures that: (i) for R^2^, larger observed values map to larger normalized scores, correctly rewarding higher explanatory power; and (ii) for MSE, RMSE, and MAE, smaller observed values (lower errors) map to larger normalized scores by reversing the direction of normalization, as shown in Eqs. 8–10. The resulting composite score ranges from 0 (worst overall performance across all four metrics) to a maximum of 1 (best overall performance). Equal weighting was assigned to all four metrics. Because min–max normalization depends on the observed minimum and maximum values, it may be sensitive to extreme metric values. However, no disproportionately extreme values were identified among the evaluated models. The robustness of the resulting rankings was further confirmed through a sensitivity analysis using alternative weighting schemes, as described in Supplementary Material (S4).

### Uncertainty analysis

Prediction uncertainty was quantified using 90% bootstrap confidence intervals (CIs) for the mean predicted Annual Maximum Daily Rainfall (AMDR) at the six standard return periods (T = 2, 5, 10, 25, 50, and 100 years). For the statistical distribution models, uncertainty was estimated using the non-parametric bootstrap resampling procedure implemented in HEC-SSP (Version 2.3.1), whereby bootstrap samples were generated by resampling the original AMDR observations with replacement, without assuming an underlying probability distribution. A total of 1000 bootstrap replications were performed, and the 90% confidence intervals were determined from the 5th and 95th percentiles of the resulting bootstrap distributions.

For the ML models, bootstrap confidence intervals were computed by repeatedly resampling the training data with replacement over 1000 iterations and calculating the mean predicted AMDR at each standard return period for each bootstrap sample. The corresponding 5th and 95th percentiles were then used to define the 90% confidence interval boundaries. For both modelling approaches, the confidence interval width (upper bound − lower bound, mm) was adopted as the primary metric for comparing predictive uncertainty among models.

## Results

Twenty-five statistical distribution models were evaluated in HEC-SSP using Annual Maximum Daily Rainfall (AMDR) data recorded at Suez meteorological station over the period 1931–2021. Return periods were computed via the Weibull plotting position formula, and 90% Bootstrap Confidence Limits were applied throughout. Goodness-of-fit was assessed using two complementary tests: the Kolmogorov–Smirnov (KS) and Anderson–Darling (AD) tests. A statistical distribution was retained only when both the KS and AD test statistics were smaller than their corresponding sample-size-dependent critical values. Of the twenty-five candidate distributions, thirteen fulfilled both criteria and were advanced to the prediction stage; the remaining twelve were excluded from further analysis. The thirteen accepted models are: Generalized Pareto (GPPM, GPMLE), Exponential (EPM, ELM, EMLE), Log-Logistic (LLPM, LLLM), Gamma (GPM, GLM), Generalized Logistic (GLLM), Generalized Extreme Value (GEVLM, GEVMLE), and Shifted Exponential (SEMLE).

Three machine learning (ML) regressors were applied: Support Vector Machine (SVM), Random Forest (RF), and XGBoost Regressor (XGBR). Each model was trained using exceedance probability as the sole predictor variable (X) and AMDR (mm) as the response variable (y). A manual 4-fold cross-validation scheme was implemented to assess generalisation performance across the full dataset of observed return periods. Prediction uncertainty was quantified using 90% Bootstrap Confidence Intervals computed for the mean prediction in each case.

### Overall model performance and ranking

**Fig. 3:**
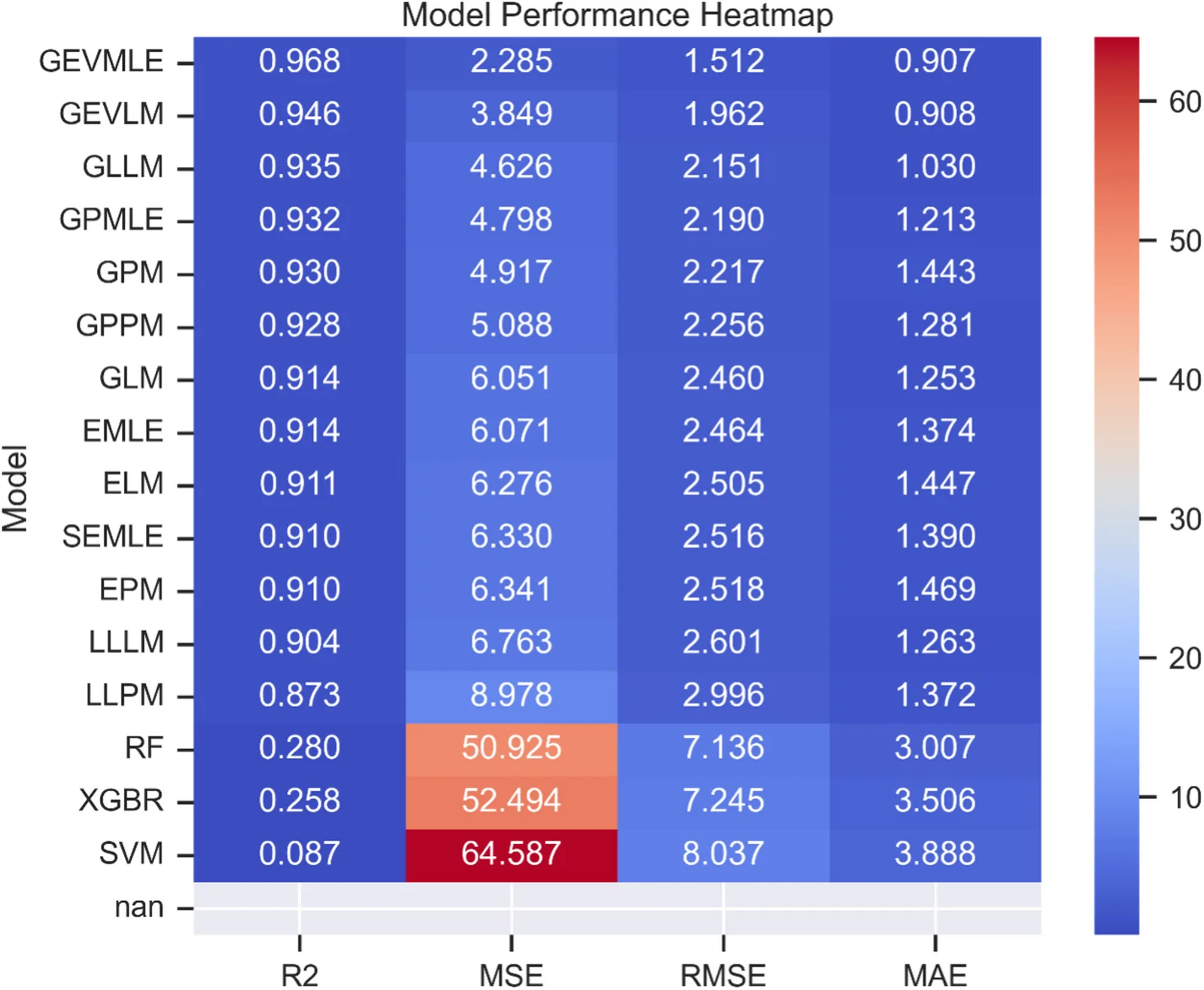
Model performance heatmap showing R^2^, MSE, RMSE, and MAE for all models.

**Fig. 4:**
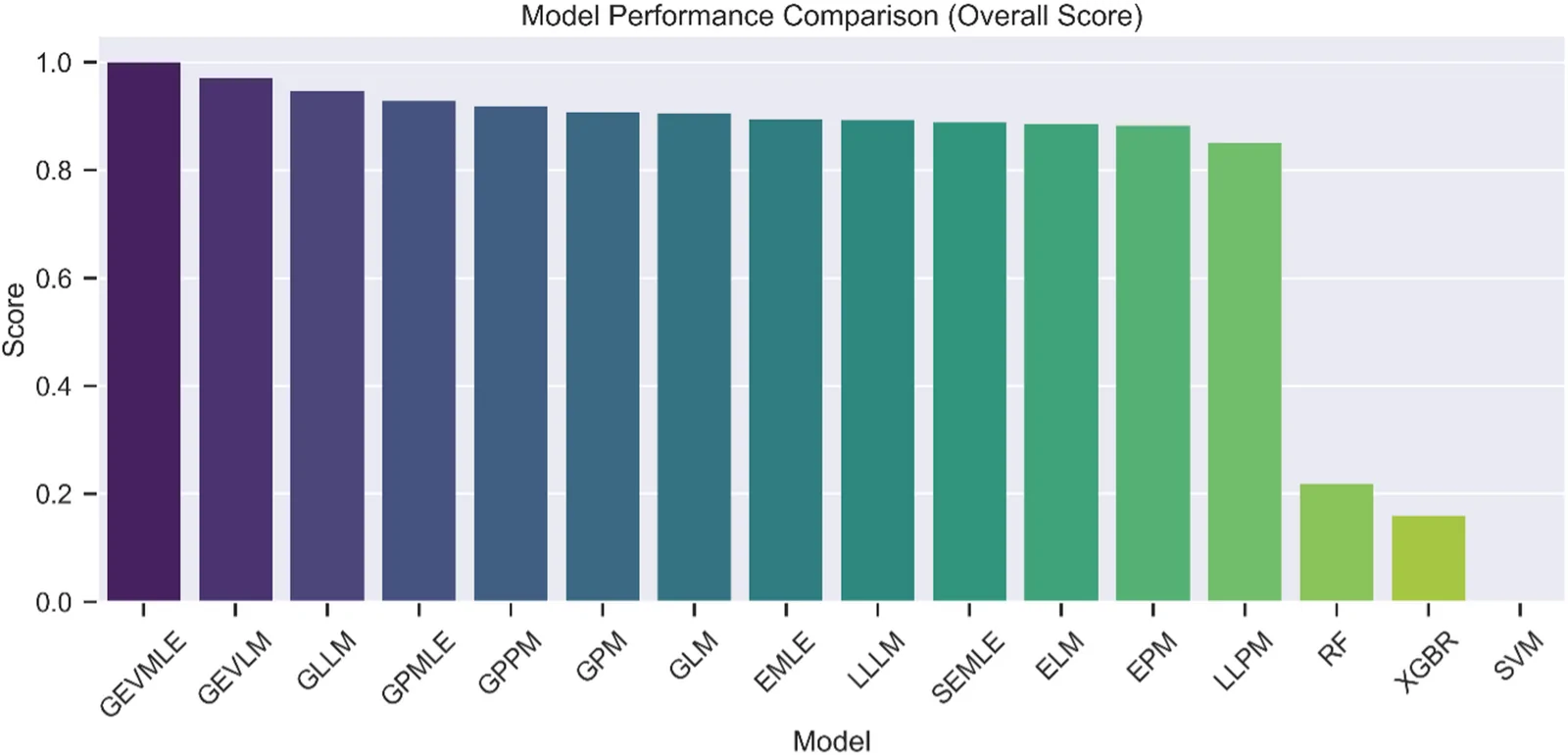
Bar chart of overall composite performance scores for all sixteen models ranked in descending order. The score was derived from normalised R^2^, MSE, RMSE, and MAE metrics.

Model performance was evaluated across all observed return periods using four statistical metrics: the coefficient of determination (R^2^), mean squared error (MSE), root mean squared error (RMSE), and mean absolute error (MAE). A composite ranking score was derived by normalising each metric to the [0, 1] range and computing a weighted aggregate, where a score of 1.000 corresponds to the best-performing model and 0 to the weakest. Complete results are presented in Table 3; the corresponding performance heatmap and comparative score charts are shown in Figs. 3 and 4.

**Table 3:** Overall model performance metrics (R^2^, MSE, RMSE, MAE) and composite ranking scores for all sixteen models evaluated. Models are ordered by composite score (descending).

Model	R^2^	MSE	RMSE	MAE	Score	Rank
**Statistical distribution models**						
GEVMLE	0.9677	2.285	1.512	0.907	1.000	1
GEVLM	0.9456	3.849	1.962	0.908	0.970	2
GLLM	0.9346	4.626	2.151	1.030	0.946	3
GPMLE	0.9322	4.798	2.190	1.213	0.928	4
GPPM	0.9281	5.088	2.256	1.281	0.918	5
GPM	0.9305	4.917	2.217	1.443	0.907	6
GLM	0.9144	6.051	2.460	1.253	0.904	7
EMLE	0.9142	6.071	2.464	1.374	0.894	8
LLLM	0.9044	6.763	2.601	1.263	0.892	9
SEMLE	0.9105	6.330	2.516	1.390	0.889	10
ELM	0.9113	6.276	2.505	1.447	0.885	11
EPM	0.9103	6.341	2.518	1.469	0.882	12
LLPM	0.8731	8.978	2.996	1.372	0.850	13
**Machine learning models**						
RF	0.2799	50.925	7.136	3.007	0.218	14
XGBR	0.2577	52.494	7.245	3.506	0.159	15
SVM	0.0867	64.587	8.037	3.888	0.000	16

All thirteen statistical distribution models demonstrated consistently high explanatory power, with R^2^ values ranging from 0.873 (LLPM) to 0.968 (GEVMLE). GEVMLE achieved the highest composite score (1.000), ranking first across all individual metrics (R^2^ = 0.968, MSE = 2.285, RMSE = 1.512 mm, MAE = 0.907 mm). The estimated location, scale, and shape parameters of the GEVMLE were 4.006, 3.766, and − 0.354, respectively. GEVLM ranked second (score = 0.970), followed by GLLM (0.946). The Log-Logistic model fitted by the method of probability-weighted moments (LLPM) returned the lowest performance among the statistical models (score = 0.850), due to its elevated error metrics, particularly at the tails of the frequency curve.

By contrast, the three ML models exhibited markedly inferior performance. R^2^ values were 0.280 (RF), 0.258 (XGBR), and 0.087 (SVM), indicating that none of the ML regressors was able to capture the distributional structure of the extreme rainfall data under the present modelling configuration. MSE values for RF, XGBR, and SVM were 50.925, 52.494, and 64.587 mm^2^ respectively, approximately 7 to 28 times larger than those of the best-performing statistical models.

### Predicted rainfall depths at standard return periods

Table 4 presents the predicted AMDR (mm) at the six standard return periods for the five highest-ranked statistical models and all three ML models. Complete predicted depths for all sixteen models are provided in Table S5 (Supplementary Material). At the 2-year return period, predictions from all models converge within a narrow range (5.00–6.00 mm), reflecting the dominance of the observed median in calibrating each model. This convergence diminishes progressively with increasing return period.

**Table 4:** Predicted AMDR (mm) at standard return periods for the five top-ranked statistical distribution models and the three machine learning models.

T (yr)	GEVMLE	GEVLM	GLLM	EPM	ELM	XGBR	RF	SVM
2	5.43	5.55	5.67	5.46	5.46	6.00	6.00	6.00
5	11.30	11.32	11.07	12.75	12.75	11.00	10.00	10.00
10	16.73	16.32	15.78	18.31	18.31	18.00	17.00	18.00
25	23.63	22.34	21.66	23.91	23.91	36.00	31.00	28.00
50	36.08	32.62	32.24	31.38	31.38	45.00	41.00	32.00
100	49.17	42.94	43.37	37.08	37.08	46.00	42.00	35.00

Top five statistical models shown by composite ranking: GEVMLE (Rank 1), GEVLM (Rank 2), GLLM (Rank 3), EPM (Rank 12), ELM (Rank 11). EPM and ELM included represent the Exponential family, which showed the most stable uncertainty profile (see Sect. 3.3).

At the 10-year return period, statistical model predictions cluster between 15.78 mm (GLLM) and 18.85 mm (LLPM), with a central tendency around 18 mm. ML models align closely with this range at T = 10 yr (SVM: 18.00 mm; RF: 17.00 mm; XGBR: 18.00 mm), which is consistent with their training data coverage. However, at longer return periods (T ≥ 25 yr), the ML models produce systematically compressed and physically unreliable estimates. At T = 100 yr, GEVMLE predicts 49.17 mm, while SVM yields only 35.00 mm and RF 42.00 mm, which fall below several statistical model estimates at the 50-year level.

Among the statistical distribution models, the Log-Logistic family (LLPM: 78.30 mm; LLLM: 74.64 mm at T = 100 yr) and models with heavy-tail parameterizations (GLLM: 43.37 mm; GEVLM: 42.94 mm) produce the highest estimates at long return periods. The Exponential family models (ELM, EMLE, EPM: 37.08 mm at T = 100 yr) and GEVMLE (49.17 mm) occupy an intermediate range.

### Uncertainty analysis

**Fig. 5:**
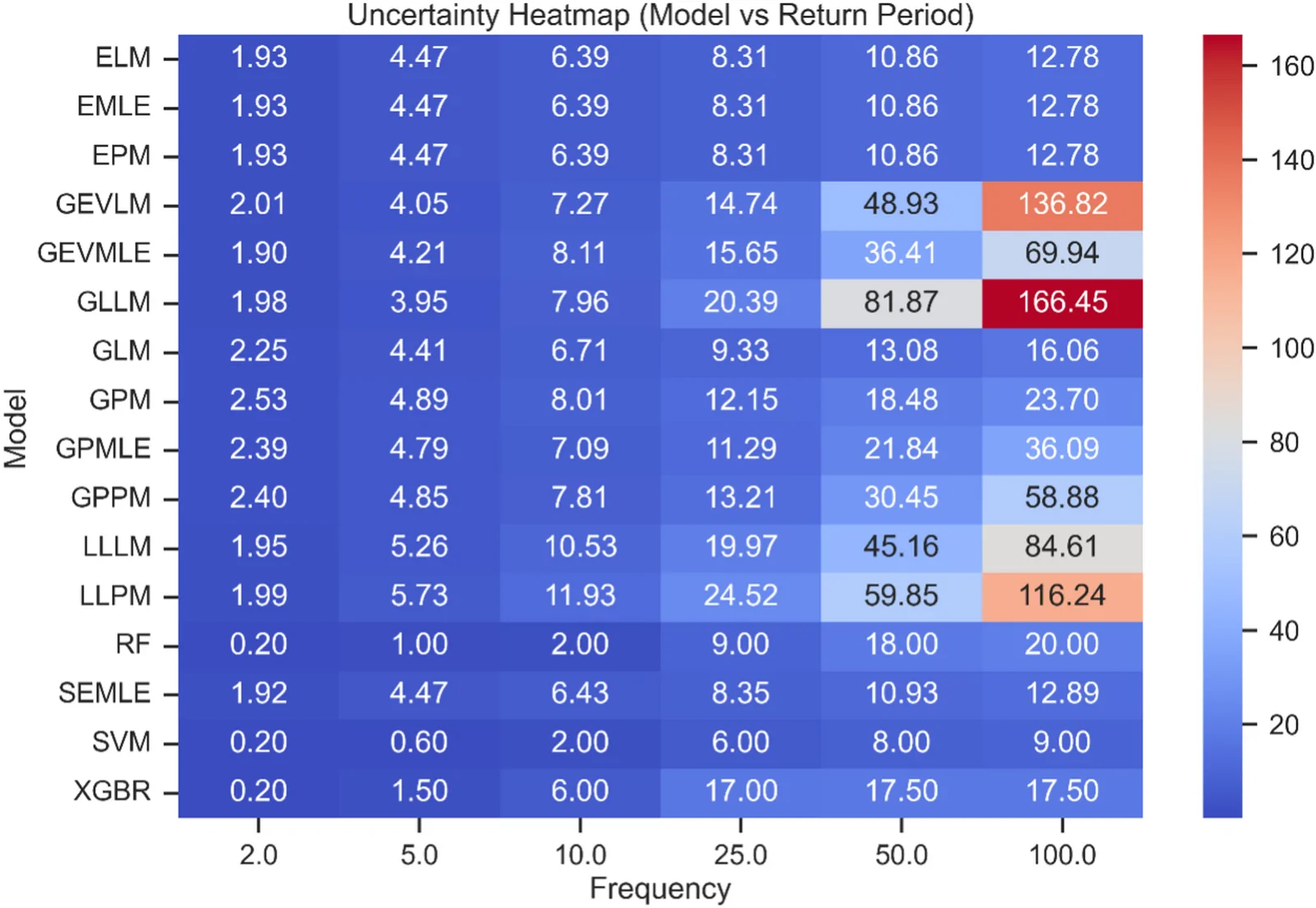
Uncertainty heatmap (90% bootstrap CI width, mm) for all models across the six standard return periods (T = 2, 5, 10, 25, 50, and 100 years).

**Fig. 6:**
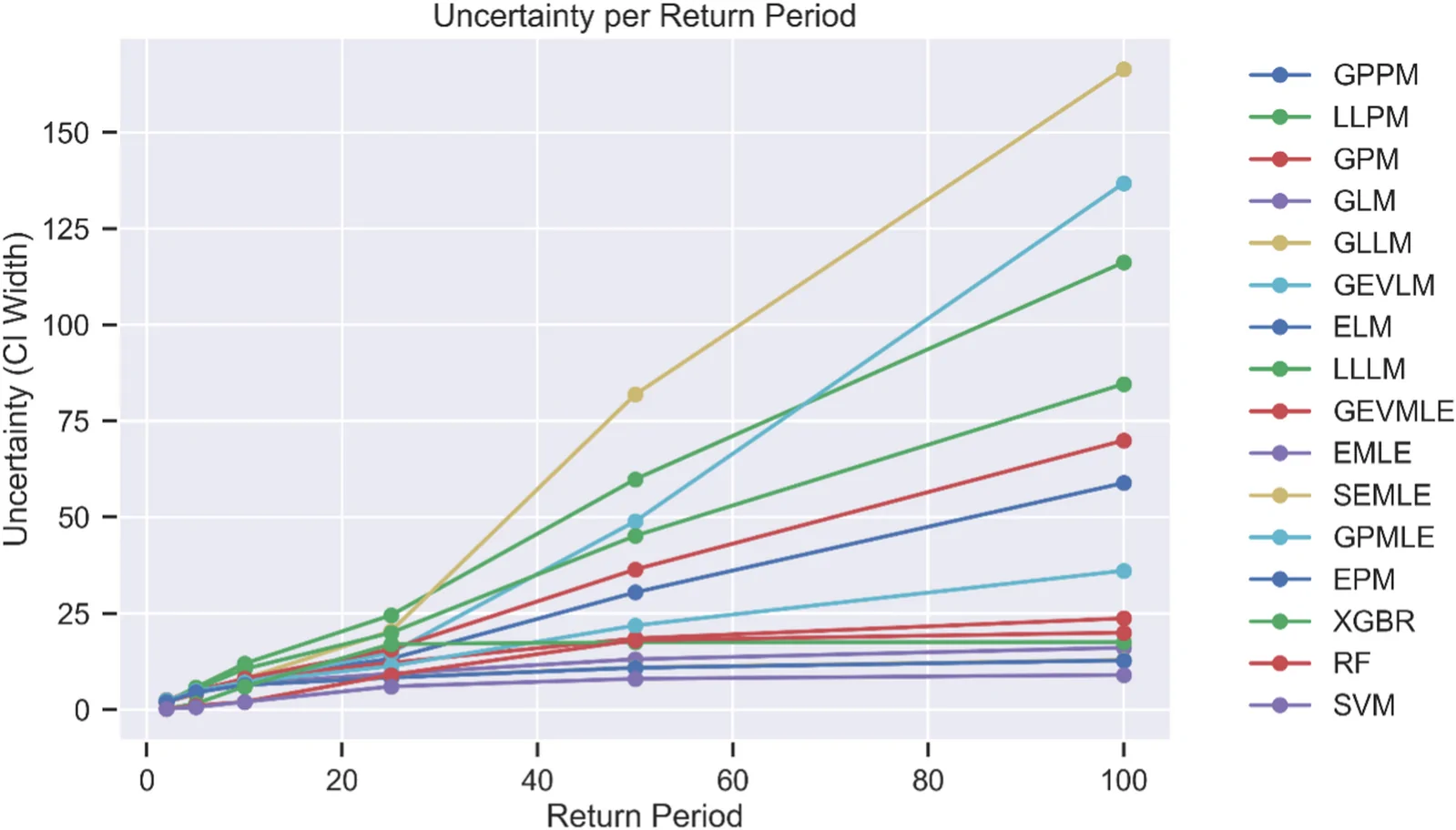
Uncertainty growth curves showing 90% CI width (mm) as a function of return period for all sixteen models. Models with heavy-tail parameterizations (GLLM, GEVLM, LLPM, LLLM) exhibit exponential CI expansion beyond T = 25 yr. Exponential family models (ELM, EMLE, EPM) and ML models show the most restrained growth.

**Fig. 7:**
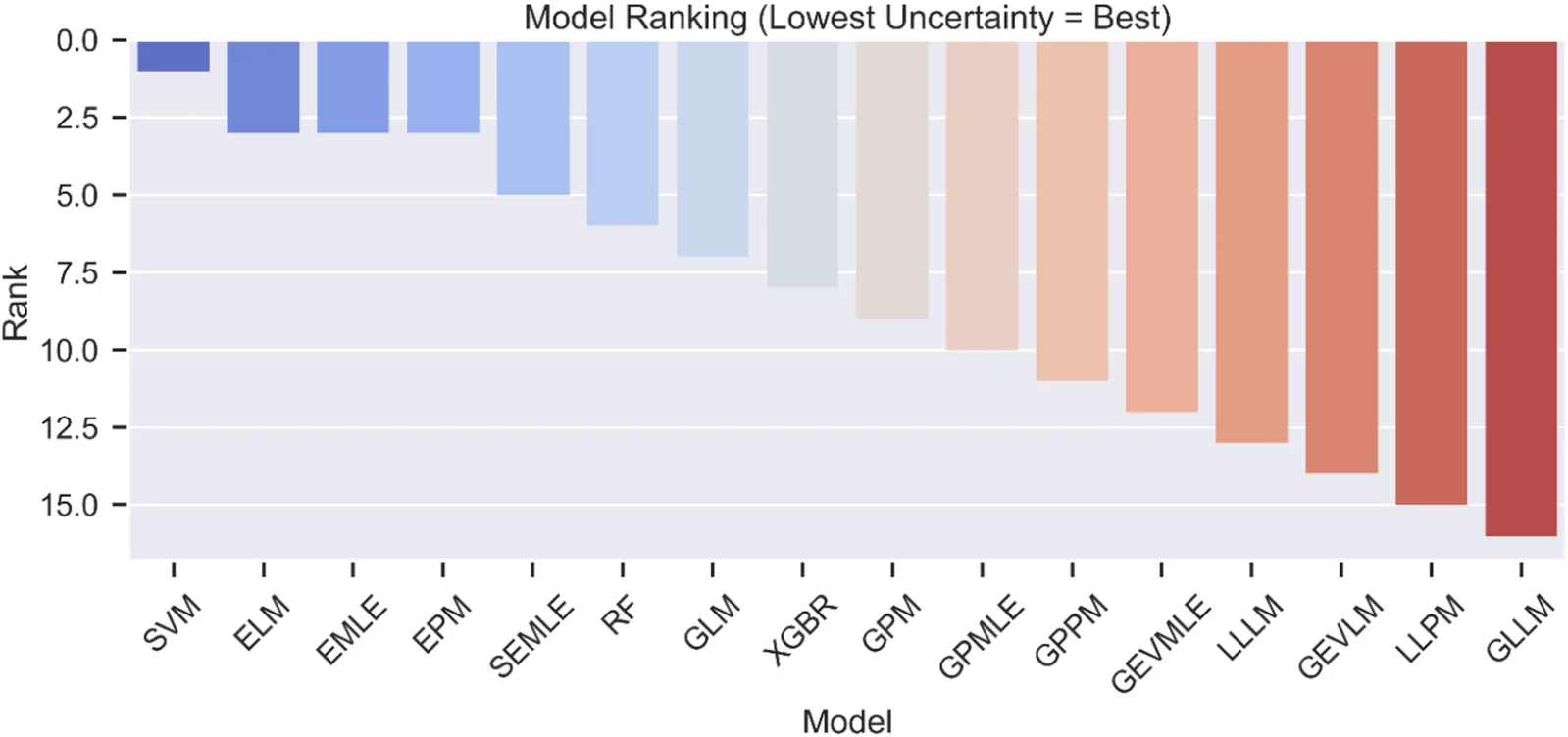
Model ranking by average uncertainty (90% CI width) across all standard return periods (lowest uncertainty = Rank 1). SVM achieves Rank 1 (narrowest CI overall), followed by ELM, EMLE, and EPM. GLLM and LLPM occupy the lowest uncertainty ranks.

The 90% Bootstrap Confidence Interval (CI) width (mm) for each model was computed at the six standard return periods (T = 2, 5, 10, 25, 50, and 100 years). Results are presented in Figs. 5, 6, 7; complete numerical values for all models are provided in Table S6 (Supplementary Material).

Among the statistical distribution models, GEVMLE returned the narrowest CI at T = 2 yr (1.90 mm, Rank- 4 overall) and maintained a comparatively stable profile through T = 25 yr (15.65 mm). However, at T = 50 yr and T = 100 yr, GEVMLE's CI expands to 36.41 mm and 69.94 mm, respectively, reflecting the increasing parametric uncertainty inherent in GEV tail estimation from a 91-year record. The Exponential family models (ELM, EMLE, EPM) exhibited the most stable uncertainty trajectories among the statistical distributions, with CI widths of 12.78 mm at T = 100 yr (Rank 3), consistent with their light-tailed parametric structure. GLLM and GEVLM showed the most severe uncertainty escalation: at T = 100 yr, GLLM reached 166.45 mm and GEVLM 136.82 mm, indicating strong sensitivity to tail parameterization under the arid-region data constraints of this study.

The ML models produced the narrowest CIs across virtually all return periods: SVM (0.20–9.00 mm), RF (0.20–20.00 mm), and XGBR (0.20–17.50 mm). As the ML models operate through interpolation within the observed exceedance probability space, their bootstrap variance is inherently small and does not capture extrapolative uncertainty.

## Discussion

### Performance of statistical distribution and machine learning models

Among the twenty-five statistical distribution models initially evaluated, thirteen satisfied both the Kolmogorov–Smirnov (KS) and Anderson–Darling (AD) goodness-of-fit criteria and were retained for subsequent analyses. The remaining twelve models were excluded because they did not adequately represent the observed AMDR series, particularly with respect to the overall distribution shape and upper-tail behavior, as demonstrated by the goodness-of-fit results presented in Table S7 (Supplementary Material). This preliminary screening ensured that only statistically acceptable models were included in the subsequent comparative evaluation.

The Generalized Extreme Value distribution fitted by Maximum Likelihood Estimation (GEVMLE) provided the most favorable overall performance among the retained statistical models. Its selection was not based solely on the highest R^2^ value (0.968), but on the combined evidence from the goodness-of-fit assessment, predictive performance metrics, return-level estimates, and uncertainty analysis. The GEV framework is theoretically appropriate for block-maxima data such as Annual Maximum Daily Rainfall and provides a flexible representation of upper-tail behavior through its shape parameter^4,11^.

The superior performance of GEV-type models is consistent with the hydrological and climatological characteristics of the Suez region. Rainfall in this arid coastal environment is dominated by infrequent, high-intensity storms. These episodic events generate highly variable annual rainfall maxima separated by prolonged dry periods, producing positively skewed distributions with pronounced upper-tail behavior. Owing to its flexible shape parameter, the GEV distribution is well suited to represent such variability and tail characteristics, making it particularly appropriate for modelling rainfall extremes in arid and semi-arid climates. Consequently, GEV-type distributions have been widely recommended for rainfall frequency analysis in regions where extreme precipitation is governed by high interannual variability and episodic storm generation^9,25,42,43^.

GEVLM (Rank 2, score = 0.970) and GLLM (Rank 3, score = 0.946) further demonstrate that L-moment estimation provides a robust alternative to Maximum Likelihood Estimation for hydrological frequency analysis. L-moment estimators are less sensitive to sampling variability and outliers^5,14,44^. This characteristic is especially relevant for the 91-year Suez record, which includes both exceptionally large rainfall events and several years with no recorded rainfall.

The competitive performance of GLLM, despite the heavier tail of the generalized logistic distribution relative to GEV, further emphasizes the importance of rigorous goodness-of-fit screening prior to model selection. Similar comparative studies have shown that no single probability distribution consistently provides the best representation under all climatic conditions, highlighting the importance of selecting models based on objective statistical evaluation rather than theoretical preference alone^3,13^.

The Log-Logistic family (LLPM, Rank13; LLLM, Rank9) exhibited the lowest predictive accuracy among the retained statistical models and the greatest expansion of confidence intervals at longer return periods. This behavior is consistent with the heavy-tailed nature of the Log-Logistic distribution, which assigns relatively greater probability to extreme rainfall events, resulting in larger estimated upper-tail quantiles and increased predictive uncertainty. From a hydrological perspective, this characteristic may be less suitable for the eastern Egyptian coastal climate, where rainfall is generated by infrequent but intense convective storms that are nevertheless constrained by limited atmospheric moisture availability^1,8^.

The three machine learning (ML) regressors (RF, XGBR, and SVM) consistently produced substantially lower performance than all retained statistical distribution models. This performance gap should be interpreted within the methodological framework adopted in the present study rather than as evidence that ML approaches are inherently unsuitable for rainfall frequency analysis. Unlike statistical frequency models, which explicitly describe the probability distribution of extreme rainfall based on extreme value theory, the ML models were trained as supervised regression algorithms using exceedance probability as the sole predictor variable. Consequently, although both approaches establish a relationship between exceedance probability and rainfall depth, they address fundamentally different modelling problems. The statistical distributions incorporate a predefined probabilistic tail structure, whereas the ML regressors learn only the empirical relationship contained within the available observations^17,21^.

The markedly lower R^2^ values obtained by the ML models therefore largely reflect the limited information content of the single-predictor framework adopted to ensure a direct and methodologically consistent comparison with statistical frequency models. Numerous hydrological studies have demonstrated that ML algorithms achieve substantially improved performance when trained using multiple climatic, atmospheric, topographic, or catchment-scale predictors^20,22,23^. Under the intentionally simplified configuration adopted here, however, the ML models lacked sufficient information to reproduce the theoretical behavior of the upper tail of the rainfall distribution, particularly beyond the range of the observed data.

This limitation becomes increasingly evident at long return periods (T ≥ 25 years), where all three ML models exhibited progressively compressed rainfall estimates. XGBR predicted nearly identical rainfall depths for the 50- and 100-year return periods, while SVM consistently produced the smallest design rainfall estimates. Such behavior reflects the well-known tendency of regression-based ML algorithms to revert toward the central tendency of the training data when extrapolating beyond the range represented by the observations, resulting in saturation of the predicted response rather than the monotonic increase expected for design rainfall quantiles. Similar behavior has been reported in previous studies evaluating ML performance for hydrological extremes^17,21,23^.

From an engineering perspective, these findings reinforce the continued importance of extreme value theory for rainfall frequency analysis under data-limited conditions. Because regulatory design practices in many arid regions remain largely based on conventional frequency analysis, the consistently strong performance of the GEV family provides additional support for its use in estimating design rainfall^4,9,25^.

### Uncertainty analysis

Although predictive accuracy is an essential indicator of model performance, reliable hydraulic design also requires robust quantification of the uncertainty associated with estimated rainfall quantiles, particularly for long return periods^45,46^.

The bootstrap analysis demonstrated distinct uncertainty characteristics between the statistical distribution models and the machine learning (ML) regressors. The ML regressors consistently produced considerably narrower confidence intervals than the statistical distribution models. This behavior should not be interpreted as indicating that the resulting confidence intervals are incorrect or inherently less reliable. Rather, it reflects the characteristics of the adopted regression framework, in which predictions are primarily governed by interpolation within the information contained in the training dataset. Instead, because the ML models were trained using exceedance probability as the sole predictor, the bootstrap resampling procedure primarily quantified variability within the observed empirical relationship and did not explicitly capture the additional uncertainty associated with probabilistic tail modelling or extrapolation beyond the historical data range.

Heavy-tailed distributions (GLLM, LLPM, LLLM) produced substantially wider confidence intervals that expanded with increasing return period, reflecting their greater sensitivity to extreme observations and upper-tail extrapolation^2,4,5^. Although such models may be appropriate when supported by sufficiently long records exhibiting pronounced heavy-tail behavior, the 91-year Suez record does not provide sufficient evidence to reliably discriminate between competing tail assumptions at extreme quantiles, particularly in arid-region datasets with high inter-annual variability^1,9^.

The moderate-tail GEV and exponential family models offer a more defensible balance between flexibility and uncertainty stability. The exponential family models (ELM, EMLE, EPM) demonstrated the most stable uncertainty profile across return periods (CI ≈ 12.78 mm at T = 100 years), reflecting their constrained single-parameter tail structure. However, their lower overall predictive performance is consistent with previous findings showing that light-tailed distributions may under-represent extreme rainfall magnitudes in regions influenced by episodic convective and cyclonic systems^13,25^.

Among all evaluated models, GEVMLE provides the best overall balance between predictive accuracy and uncertainty under the adopted statistical framework. This behavior reflects the flexibility of the three-parameter GEV structure, which allows sufficient tail adaptation to observed extremes without introducing the excessive tail sensitivity characteristic of logistic-based distributions. These findings are consistent with regional frequency analyses in arid and semi-arid environments, where GEV-type models are frequently identified as effective compromise solutions between bias and uncertainty in extreme rainfall estimation^4,9,25^.

The substantial uncertainty associated with return periods of 50–100 years, even for the best-performing statistical models, highlights the inherent difficulty of estimating rare rainfall extremes from single-station records. Although the 91-year AMDR series is relatively long by regional standards, it remains insufficient to fully constrain upper-tail behavior with high confidence, as reflected by the relatively wide confidence intervals. These findings highlight the importance of extending observational records and exploring regional frequency analysis or regional pooling approaches to improve the reliability of long-return-period rainfall estimates in data-scarce arid catchments.

The negative shape parameter (− 0.354) indicates a bounded upper-tail form of the fitted GEVMLE distribution, which influences the estimated upper-tail quantiles at long return periods^12^. Therefore, although GEVMLE provides the best overall performance among the evaluated models under the adopted framework, its estimates at 50- and 100-year return periods should be interpreted together with their wide confidence intervals rather than as precise predictions of rare rainfall events.

It is important to distinguish between a model-based return-level estimate and an independently validated design rainfall value. Although the 100-year return level can be estimated from a 91-year annual maximum record through frequency analysis, it necessarily involves extrapolation beyond the observed record. Therefore, the GEVMLE estimate of 49.17 mm should be interpreted as a model-based estimate of the 100-year return level rather than as a precisely known design rainfall. The substantial 90% confidence-interval width (69.94 mm) further demonstrates the uncertainty associated with this extrapolation. Accordingly, the 50- and 100-year estimates should be interpreted with considerable caution, particularly when used for engineering design, and should ideally be complemented by longer records or regional frequency analysis.

The estimated AMDR return levels are applicable to daily-scale rainfall assessment, including hydrological analysis and preliminary flood-risk evaluation. However, they should not be directly used as substitutes for duration-specific IDF design rainfall, which requires sub-daily rainfall data and duration-based frequency analysis.

### Limitations

The ML models were developed using exceedance probability as the sole predictor variable. This simplified configuration was intentionally adopted to ensure a direct, controlled, and methodologically consistent comparison with conventional statistical frequency models, which likewise establish the relationship between exceedance probability and rainfall magnitude. Therefore, the comparatively lower performance of the ML models should be interpreted within the scope of this experimental framework rather than as evidence that ML approaches are inherently unsuitable for rainfall frequency analysis.

The use of a single predictor was primarily dictated by the available dataset, which consists of a homogeneous 91-year AMDR record (1931–2021). Incorporating additional climatic, atmospheric, or catchment-scale predictors was not feasible because long-term datasets with comparable temporal coverage and consistent quality were unavailable. Integrating external datasets with different temporal resolutions or observation periods would have reduced data consistency and compromised the methodological comparability between the statistical frequency models and the ML approaches.

Furthermore, the analysis was based on observations from a single long-term rainfall station located in an arid coastal environment. Although the Suez record represents one of the longest and most reliable AMDR datasets currently available in Egypt, caution should be exercised when extrapolating the findings to other climatic or physiographic settings. Future studies incorporating multiple stations across diverse arid and semi-arid regions, together with regional frequency analysis and richer predictor datasets, would provide a more comprehensive assessment of the transferability and general applicability of the proposed comparative framework.

### Conclusions and recommendations

This study presented a comprehensive and systematic comparison of twenty-five statistical distribution models and three machine learning (ML) regressors — Support Vector Machine (SVM), Random Forest (RF), and XGBoost Regressor (XGBR) — for Annual Maximum Daily Rainfall (AMDR) frequency analysis at Suez meteorological station, Egypt, using a 91-year observational record (1931–2021). Models were evaluated in terms of predictive accuracy and uncertainty quantification using 90% bootstrap confidence intervals, with a composite ranking framework enabling objective cross-paradigm comparison.

The application of dual goodness-of-fit criteria (KS and AD) resulted in the elimination of twelve statistical distribution models, ensuring that only statistically consistent models were retained for further analysis. All retained statistical distribution models significantly outperformed the ML regressors. Specifically, R^2^ values for statistical models ranged from 0.873 to 0.968, whereas ML models (SVM, RF, XGBR) achieved substantially lower performance (0.087–0.280), confirming the superiority of classical extreme value distributions under a single-predictor exceedance-probability framework.

Among all models, the Generalized Extreme Value distribution fitted by maximum likelihood (GEVMLE) emerged as the best-performing model, achieving the highest composite score (1.000) and ranking first across all performance metrics (R^2^ = 0.968, RMSE = 1.512 mm). This result is consistent with extreme value theory, which identifies the GEV distribution as the limiting form for block maxima. The strong performance of GEVLM and GLLM further highlights the robustness of L-moment estimation techniques in arid-region rainfall frequency analysis.

At high return periods (T ≥ 25 years), ML models systematically underestimated extreme rainfall magnitudes relative to statistical models, reflecting their limited ability to extrapolate beyond the observed exceedance probability domain. This limitation becomes particularly critical in engineering design applications where long-return-period estimates are required.

Uncertainty analysis revealed a clear trade-off between tail flexibility and stability. Heavy-tailed models (GLLM, GEVLM, LLPM, LLLM) produced the widest confidence intervals (up to 166 mm at T = 100 years), indicating high sensitivity to tail assumptions under limited data length. In contrast, Exponential-family models showed narrow uncertainty bounds but reduced ability to capture extreme events. GEVMLE provided the most balanced performance, combining the highest accuracy with moderate uncertainty (CI = 69.94 mm at T = 100 years).

Overall, the findings demonstrate that for long-term AMDR records in arid environments, statistically based extreme value models remain more reliable than ML regressors when input information is limited to exceedance probability. It is anticipated that the findings and the methodological framework presented here will support evidence-based model selection in operational hydrology and contribute to safer, more reliable hydraulic infrastructure design in water-scarce and flood-prone arid regions.

### Recommendations

1. Machine learning (ML) regressors should not be used as standalone tools for rainfall frequency analysis in engineering design when the predictor space is limited to exceedance probability, particularly for return periods exceeding T = 10 years. Under this configuration, SVM, RF, and XGBR exhibit compressed tail estimates and confidence intervals that do not reflect physically consistent extrapolation behavior. This limitation is specific to single-variable ML implementations; therefore, hybrid or multi-feature ML frameworks incorporating physically meaningful climatic and hydrological predictors may offer improved performance and should be investigated in future studies.
2. Uncertainty quantification should be considered an essential component of rainfall frequency analysis rather than an optional addition. The results demonstrate substantial uncertainty at long return periods (T ≥ 50 years) across all statistical models, including the best-performing GEVMLE (e.g., CI = 36.41 mm at T = 50 years), highlighting the irreducible nature of uncertainty in arid-region frequency estimation using finite-length records. Accordingly, uncertainty bounds should be explicitly incorporated into engineering design, risk assessment, and safety factor selection to avoid overconfidence in point estimates.

### Future research

The findings of this study suggest several promising directions for future research aimed at improving rainfall frequency analysis in arid and semi-arid regions.

(i) Hybrid statistical–machine learning frameworks: future work should explore hybrid and ensemble approaches that combine the robust tail behavior of parametric extreme value distributions with the flexibility of machine learning (ML) methods. Methods such as distributional neural networks, quantile regression forests, and physics-informed ML models, particularly when trained on enriched predictor sets, may enhance the ability to model non-linear dependencies and improve estimation of rare extreme events.
(ii) Regional-scale validation and transferability: the replication of the present methodological framework across multiple arid and semi-arid stations in North Africa and the Middle East is recommended. Such comparative studies would allow assessment of spatial robustness and model transferability and would support the development of regionally consistent guidelines for extreme rainfall frequency analysis in infrastructure design under data-scarce conditions.

## Supplementary Information

Below is the link to the electronic supplementary material.

Supplementary Material 1

## Data Availability

Available on request.

## References

[1] Arnous, M. O., El-Rayes, A. E., El-Nady, H. & Helmy, A. M. Flash flooding hazard assessment, modeling, and management in the coastal zone of Ras Ghareb City, Gulf of Suez, Egypt. *J. Coast. Conserv.* **26**(6), 77 (2022).

[2] Ciupak, M., Ozga-Zieliński, B., Tokarczyk, T. & Adamowski, J. A probabilistic model for maximum rainfall frequency analysis. *Water* **13**(19), 2688 (2021).

[3] Yong, S. L. S., Ng, J. L., Huang, Y. F. & Ang, C. K. Assessment of the best probability distribution method in rainfall frequency analysis for a tropical region. *Malays. J. Civ. Eng.* **33**(1) (2021).

[4] Back, Á. J. & Bonfante, F. M. Evaluation of generalized extreme value and Gumbel distributions for estimating maximum daily rainfall. *Rev. Bras. Cienc. Ambient.* **56**(4), 654–664 (2021).

[5] Billios, M. & Vasiliades, L. Regional frequency analysis of annual maximum rainfall and sampling uncertainty quantification. *Environ. Earth Sci. Proc.* **32**(1), 3 (2025).

[6] Khan, Z., Rahman, A. & Karim, F. An assessment of uncertainties in flood frequency estimation using bootstrapping and Monte Carlo simulation. *Hydrology* **10**(1), 18 (2023).

[7] Elbeih, S. F., Hagage, M., Attia, W. & Abd el-sadek, E. Using treated wastewater in groundwater recharge at wadi el farigh area, Egypt: GIS and remote sensing applications. In *International Conference of Remote Sensing and Space Sciences Applications* . 63–71. (Springer Nature Switzerland, 2022).

[8] Mansour, M. M., Nasr, M., Fujii, M., Yoshimura, C. & Ibrahim, M. G. Evaluation of a reliable method for flash flood hazard mapping in arid regions: a case study of the Gulf of Suez, Egypt. In *International Conference on Environment Science and Engineering*. 103–117. (Springer Nature Singapore, 2022).‏

[9] Mengistu, T. D. et al. Regional flood frequency analysis for sustainable water resources management of Genale–Dawa River Basin, Ethiopia. *Water* **14**(4), 637 (2022).

[10] Zheng, W., Liu, S. & Zhou, Z. Rainfall frequency analysis: Interval estimations for Weibull distribution based on specially formulated pivotal quantities. *Theor. Appl. Climatol.* **157**(1), 61 (2026).

[11] Abdulali, B. A. A., Abu Bakar, M. A., Ibrahim, K. & Mohd Ariff, N. Extreme value distributions: An overview of estimation and simulation. *J. Probab. Stat.* **2022**(1), 5449751 (2022).

[12] Balacumaresan, H., Hossain, I. & Imteaz, M. A. Climate change impacts on design rainfall extremes employing empirical disaggregation-based rainfall intensities: A case study for East Melbourne. *Sustain. Water Resour. Manag.* **11**(6), 134 (2025).

[13] Montes-Pajuelo, R., Rodríguez-Pérez, Á. M., López, R. & Rodríguez, C. A. Analysis of probability distributions for modelling extreme rainfall events and detecting climate change: Insights from mathematical and statistical methods. *Mathematics* **12**(7), 1093 (2024).

[14] Agbonaye, A. Comparison of L-moment and method of moments as parameter estimators for identification and choice of the most appropriate rainfall distribution models for design of hydraulic structures. *J. Civ. Eng. Sci. Technol.* 10.33736/jcest.4207.2022 (2022).

[15] Hossain, I. et al. Comparison of estimation techniques for generalised extreme value (GEV) distribution parameters: A case study with Tasmanian rainfall. *Int. J. Environ. Sci. Technol.* **19**(8), 7737–7750 (2022).

[16] Zishen, C. H. E. N. & Zengmei, L. I. U. Comparative analysis of parameter estimation methods of generalized extreme value distribution. *Acta Sci. Natur. Univ. Sunyatseni* **49**(6), 105–109 (2023).

[17] Afandi, G. E., Moustafa, A., Ibrahim, S. & Irfan, M. An in-depth analysis of approaches for forecasting extreme rainfall events leveraging artificial intelligence, machine learning, and deep learning techniques. *Earth Syst. Environ*. 1–32 (2025).‏

[18] Al-Wardy, M., Zarei, E., Nikoo, M. R. & Al-Rawas, G. Toward enhanced coastal flooding forecasting using deterministic and probabilistic models. *Nat. Hazards* **122**(10), 453 (2026).

[19] Hagage, M., Abdulaziz, A. M., Hewaidy, A. G. A. & Shetaia, S. A. Unveiling the past: Utilizing satellite imagery archives to study archaeological landscapes in the northeastern Nile Delta, Egypt. *Anthropocene* **44**, 100409 (2023).

[20] Hassan, D., Isaac, G. A., Taylor, P. A. & Michelson, D. Optimizing radar-based rainfall estimation using machine learning models. *Remote Sens.* **14**(20), 5188 (2022).

[21] Herath, H. M. V. V., Chadalawada, J. & Babovic, V. Hydrologically informed machine learning for rainfall–runoff modelling: Towards distributed modelling. *Hydrol. Earth Syst. Sci.* **25**(8), 4373–4401 (2021).

[22] Pinthong, S. et al. Imputation of missing monthly rainfall data using machine learning and spatial interpolation approaches in Thale Sap Songkhla River Basin, Thailand. *Environ. Sci. Pollut. Res.* **31**(41), 54044–54060 (2024). 10.1007/s11356-022-23022-8 36173524

[23] Singh, A. K. et al. An integrated statistical-machine learning approach for runoff prediction. *Sustainability* **14**(13), 8209 (2022).

[24] Hinis, M. A., Yurekli, K. & Erdogan, M. Establishing regional intensity-duration-frequency (IDF) relationships by using the L-moment approach and genetically based techniques for the Euphrates-Tigris basin. *Theor. Appl. Climatol.* **155**(2), 1363–1380 (2024).

[25] Prahadchai, T., Busababodhin, P. & Park, J. S. Regional flood frequency analysis of extreme rainfall in Thailand, based on L-moments. *Commun. Stat. Appl. Methods* **31**(1), 37–53 (2024).

[26] Shokouhifar, Y., Lotfirad, M., Esmaeili-Gisavandani, H. & Adib, A. Evaluation of climate change effects on flood frequency in arid and semi-arid basins. *Water Supply* **22**(8), 6740–6755 (2022).

[27] Ershad, M., Keshtkar, A., Hosseini, S. M. & Afzali, A. Analysis of temporal trend of groundwater quality using nonparametric Mann-Kendall and Sen’s methods (case study: Yazd-Ardakan Plain). *Geogr. Environ. Plan.* **32**(4), 87–106 (2021).

[28] Sun, F., Roderick, M. L. & Farquhar, G. D. Rainfall statistics, stationarity, and climate change. *Proc. Natl. Acad. Sci. U. S. A.* **115**(10), 2305–2310 (2018). 29463723 10.1073/pnas.1705349115 PMC5878000

[29] Getahun, A., Raju, U. J. P. & Yirga, G. Statistical analysis of the return period and probability distribution of annual maximum rainfall in southern Ethiopia. *J. Water Clim. Change* **17**(2), 403–425 (2026).

[30] Wehner, M. F. et al. On the uncertainty of long-period return values of extreme daily precipitation. *Front. Clim.* **6**, 1343072 (2024).

[31] Li, M. et al. Application of L-moment method for regional frequency analysis of meteorological drought across the Loess Plateau, China. *PLoS ONE* **17**(9), e0273975 (2022). 36048864 10.1371/journal.pone.0273975 PMC9436156

[32] Rahayu, D. & Chumaedi, I. Utilization of HEC DSS and HEC SSP software for rainfall frequency analysis in drainage system design for city’s industrial area. *J. Tek. Sipil Lingkungan*10.29244/jsil.10.1.179-192 (2025).

[33] Zeng, X., Wang, D. & Wu, J. Evaluating the three methods of goodness of fit test for frequency analysis. *J. Risk Anal. Crisis Response* **5**(3), 178–187 (2015).

[34] Hu, D., Yu, X. & Wang, J. Statistical inference in rough set theory based on Kolmogorov-Smirnov goodness-of-fit test. *IEEE Trans. Fuzzy Syst.* **25**(4), 799–812 (2016).

[35] Shin, H., Jung, Y., Jeong, C. & Heo, J. H. Assessment of modified Anderson-Darling test statistics for the generalized extreme value and generalized logistic distributions. *Stoch. Environ. Res. Risk Assess.* **26**(1), 105–114 (2012).

[36] Marchev, Jr, A., & Marchev, V. Automated algorithm for multi-variate data synthesis with Cholesky decomposition. In *Proceedings of the 7th International Conference on Algorithms, Computing and Systems*. 1–6 (2023).‏

[37] Yürekli, K., Kurunç, A. & Öztürk, F. Testing the residuals of an ARIMA model on the Cekerek stream watershed in Turkey. *Turk. J. Eng. Environ. Sci.* **29**(2), 61–74 (2005).

[38] Masood, A., Niazkar, M., Zakwan, M. & Piraei, R. A machine learning-based framework for water quality index estimation in the Southern Bug River. *Water* **15**(20), 3543 (2023).

[39] Srijan, R. N. et al. Machine learning-based forecasting of tidal river water levels: A case study on the Halda River, Bangladesh. In *Proceedings of International Conference on Civil Engineering Research & Innovations*. Vol. 12. 14 (2025).‏

[40] Mao, G. et al. Comprehensive comparison of artificial neural networks and long short-term memory networks for rainfall-runoff simulation. *Phys. Chem. Earth Parts A/B/C* **123**, 103026 (2021).

[41] Chicco, D., Warrens, M. J. & Jurman, G. The coefficient of determination R-squared is more informative than SMAPE, MAE, MAPE, MSE and RMSE in regression analysis evaluation. *PeerJ Comput. Sci.* **7**, e623 (2021). 34307865 10.7717/peerj-cs.623 PMC8279135

[42] Benaini, M., Achite, M., Amin, M. M. & Singh, V. P. Frequency analysis of annual maximum daily precipitation in northeastern Algeria: Mapping and implications under climate variability. *Theor. Appl. Climatol.* **153**(3), 1411–1424 (2023).

[43] Gado, T. A. et al. Event-based rainfall analysis in Sinai, Egypt. *Hydrol. Sci. J.* **69**(5), 622–638 (2024).

[44] Ishaq, F. Application of Bayesian techniques and L-moments I regional frequency analysis of rainfall extremes: Insight from Northern Pakistan. *Eksplorium-Bull. Pusat Teknol. Bahan Galian Nuklir* **46**(1), 1755–1770 (2025).

[45] Maity, R. Frequency analysis, risk and uncertainty in hydroclimatic analysis. In *Statistical Methods in Hydrology and Hydroclimatology* 141–184 (Springer Nature Singapore, 2022).

[46] Ouarda, T. B., Charron, C. & St-Hilaire, A. Uncertainty of stationary and nonstationary models for rainfall frequency analysis. *Int. J. Climatol.* **40**(4), 2373–2392 (2020).

