# Supplementary material for: Rainfall frequency and uncertainty analysis in arid regions using machine learning and statistical distribution models based on a 91-year rainfall record
Source: Sci Rep. 2026 Sep 15;16:28765. doi: 10.1038/s41598-026-68681-6 (PMC13578317; doi:10.1038/s41598-026-68681-6)
Supplement: Supplementary file 1 [file 41598_2026_68681_MOESM1_ESM.docx]

*Supplementary Material*

Rainfall Frequency and Uncertainty Analysis in Arid Regions Using Machine Learning and Statistical Distribution Models Based on a 91-Year Rainfall Record

Mohammed Hagage ^(1) *^

^1)^ Engineering Applications and Water Division, National Authority for Remote Sensing and Space Sciences (NARSS), El-Nozha El-Gedida, P.O. Box: 1564, Cairo, Egypt.

* Corresponding author email. [mohammed.hagage@narss.sci.eg](mailto:mohammed.hagage@narss.sci.eg)

S1. Complete Annual Maximum Daily Rainfall Dataset

Table S1 presents the complete AMDR series for Suez meteorological station over the 91-year period 1931–2021. Values highlighted in yellow correspond to years with notably elevated AMDR (≥ 16.0 mm) that represent high-magnitude events in this hyper-arid record. Three years (1974, 1975, 1995) recorded zero AMDR, reflecting years in which no measurable daily rainfall event was recorded at the station.

Table S1: Annual Maximum Daily Rainfall (AMDR, mm) at Suez meteorological station, 1931–2021.

| Year | AMDR (mm) | Year | AMDR (mm) | Year | AMDR (mm) |
| --- | --- | --- | --- | --- | --- |
| 1931 | 8.5 | 1962 | 2.4 | 1992 | 5.6 |
| 1932 | 1 | 1963 | 3 | 1993 | 0.4 |
| 1933 | 4 | 1964 | 10 | 1994 | 7.7 |
| 1934 | 2 | 1965 | 49.6 | 1995 | 0 |
| 1935 | 0.5 | 1966 | 3.6 | 1996 | 2.6 |
| 1936 | 5.5 | 1967 | 22 | 1997 | 8.5 |
| 1937 | 10.5 | 1968 | 2.8 | 1998 | 2.8 |
| 1938 | 7.5 | 1969 | 2.7 | 1999 | 2.6 |
| 1939 | 32.5 | 1970 | 1.4 | 2000 | 3.8 |
| 1940 | 11 | 1971 | 9.3 | 2001 | 2 |
| 1941 | 7.5 | 1972 | 5.2 | 2002 | 3.4 |
| 1942 | 11.5 | 1973 | 3.8 | 2003 | 6 |
| 1943 | 12 | 1974 | 0 | 2004 | 4.3 |
| 1944 | 24.5 | 1975 | 0 | 2005 | 7.9 |
| 1945 | 12.5 | 1976 | 9 | 2006 | 3 |
| 1946 | 5.5 | 1977 | 9.2 | 2007 | 0.8 |
| 1947 | 2.5 | 1978 | 4.8 | 2008 | 1.6 |
| 1948 | 12 | 1979 | 6.8 | 2009 | 3.9 |
| 1949 | 1 | 1980 | 5.2 | 2010 | 4 |
| 1950 | 3 | 1981 | 6.7 | 2011 | 10.2 |
| 1951 | 10 | 1982 | 7.7 | 2012 | 0.1 |
| 1952 | 30 | 1983 | 6.3 | 2013 | 6.6 |
| 1953 | 10 | 1984 | 9.7 | 2014 | 6.2 |
| 1954 | 9 | 1985 | 16.3 | 2015 | 18.9 |
| 1955 | 6 | 1986 | 4.4 | 2016 | 16 |
| 1956 | 7 | 1987 | 9.7 | 2017 | 1 |
| 1957 | 5 | 1988 | 16.5 | 2018 | 2.8 |
| 1958 | 2.1 | 1989 | 0.3 | 2019 | 11.4 |
| 1959 | 7.9 | 1990 | 22 | 2020 | 44.1 |
| 1960 | 2.3 | 1991 | 10.5 | 2021 | 5.2 |
| 1961 | 8 |  |  |  |  |

S2. HEC-SSP Configuration Settings

Table S2 summarizes the complete configuration settings applied in HEC-SSP (version 2.3.1) for statistical distribution fitting, goodness-of-fit testing, return period estimation, and confidence interval computation.

Table S2: HEC-SSP (version 2.3.1) configuration settings used for all statistical distribution model analyses in this study.

| Setting | Value / Description |
| --- | --- |
| Software | HEC-SSP Version 2.3.1 |
| Analysis type | Annual Maximum Series (AMS) |
| Plotting position | Weibull: P = i / (n + 1) |
| Confidence interval method | Bootstrap (non-parametric resampling) |
| Bootstrap replications | 1,000 |
| Confidence level | 90% (5th–95th percentile) |
| Goodness-of-fit tests | Kolmogorov–Smirnov (KS) and Anderson–Darling (AD) |
| KS retention threshold | < 0.10 |
| AD retention threshold | < 1.00 |
| Standard return periods | T = 2, 5, 10, 25, 50, 100 years |
| Output | Expected AMDR (mm) and 90% CI bounds at each return period |

S3. Four-Fold Cross-Validation Design for Machine Learning Models

The 91-year AMDR dataset was partitioned into four temporally consecutive folds of approximately equal size for cross-validation of the three ML regressors.

Table S3: Four-fold cross-validation design for ML model training and evaluation. Each fold uses ~23 years as the test set and the remaining ~68 years as the training set. Performance metrics (R², MSE, RMSE, MAE) were computed for each fold independently. Support Vector Machine (SVM), Random Forest (RF), and XGBoost Regressor (XGBR).

| Fold | R2(RF) | MSE(RF) | RMSE(RF) | MAE(RF) | MAPE(RF) | R2(SVM) | MSE(SVM) | RMSE(SVM) | MAE(SVM) | MAPE(SVM) | R2(XGBR) | MSE(XGBR) | RMSE(XGBR) | MAE(XGBR) | MAPE(XGBR) |
| --- | --- | --- | --- | --- | --- | --- | --- | --- | --- | --- | --- | --- | --- | --- | --- |
| 0 | -0.66818 | 197.0104 | 14.03604 | 8.883218 | 34.51789 | -0.83575 | 216.7998 | 14.72412 | 8.990279 | 33.16738 | -0.65538 | 195.4984 | 13.98207 | 8.797702 | 33.93162 |
| 1 | 0.067132 | 1.220558 | 1.104789 | 0.96941 | 12.35707 | -0.6041 | 2.098796 | 1.448722 | 1.320637 | 17.90252 | -3.74889 | 6.213416 | 2.492672 | 2.214729 | 31.2879 |
| 2 | 0.312617 | 0.669711 | 0.818359 | 0.732826 | 16.82819 | 0.672956 | 0.318636 | 0.564478 | 0.489398 | 10.07076 | -2.63718 | 3.543668 | 1.882463 | 1.602928 | 42.45178 |
| 3 | -2.28297 | 2.701872 | 1.643737 | 1.370721 | 297.6198 | -45.1409 | 37.9738 | 6.162289 | 4.791783 | 1153.636 | -2.09706 | 2.548864 | 1.596516 | 1.313723 | 290.2719 |
| Overall | 0.279902 | 50.92479 | 7.13616 | 3.006828 | 88.05283 | 0.086712 | 64.58704 | 8.036606 | 3.888203 | 294.3542 | 0.257713 | 52.49396 | 7.245271 | 3.506101 | 97.38925 |

S4. Sensitivity of Model Rankings to Composite Score Weighting

The composite normalized ranking score presented in the main manuscript assigns equal weight (0.25) to each of the four-performance metrics (R², MSE, RMSE, MAE). To assess the robustness of the model rankings to this weighting assumption, three alternative weighting schemes were evaluated:

i. R²-dominant weighting
ii. (R² = 0.40; MSE = RMSE = MAE = 0.20): Assigns greater weight to overall explanatory power.
iii. Error-dominant weighting (RMSE = 0.40; R² = MSE = MAE = 0.20): Assigns greater weight to root-mean-square error, which penalizes large individual deviations more severely.
iv. MAE-dominant weighting (MAE = 0.40; R² = MSE = RMSE = 0.20): Assigns greater weight to mean absolute error, which is less sensitive to outliers than RMSE.

Under all three alternative weighting schemes, GEVMLE retained Rank 1 and SVM retained Rank 16 (worst). Minor rank changes (±1–2 positions) were observed for mid-ranked models (Ranks 4–12), with no systematic reordering between the statistical distribution models and the ML regressors. These results confirm that the principal conclusions of the study are robust to the choice of weighting scheme within the composite score framework.

S5. Predicted Annual Maximum Daily Rainfall (AMDR, mm) at Standard Return Periods

Table S5a: Predicted AMDR (mm) for the thirteen retained statistical distribution models at standard return periods (T = 2, 5, 10, 25, 50, and 100 years).

| T (yr) | GPPM | LLPM | GPM | GLM | GLLM | GEVLM | ELM | LLLM | GEVMLE | EMLE | SEMLE | GPMLE | EPM |
| --- | --- | --- | --- | --- | --- | --- | --- | --- | --- | --- | --- | --- | --- |
| 2 | 5.00 | 5.23 | 5.20 | 5.55 | 5.67 | 5.55 | 5.46 | 5.23 | 5.43 | 5.46 | 5.49 | 5.32 | 5.46 |
| 5 | 12.85 | 11.72 | 12.79 | 12.68 | 11.07 | 11.32 | 12.75 | 11.57 | 11.30 | 12.75 | 12.70 | 12.55 | 12.75 |
| 10 | 18.56 | 18.85 | 18.74 | 18.10 | 15.78 | 16.32 | 18.31 | 18.46 | 16.73 | 18.31 | 18.19 | 18.18 | 18.31 |
| 25 | 24.36 | 29.30 | 24.87 | 23.57 | 21.66 | 22.34 | 23.91 | 28.46 | 23.63 | 23.91 | 23.73 | 24.01 | 23.91 |
| 50 | 32.33 | 51.43 | 33.24 | 30.93 | 32.24 | 32.62 | 31.38 | 49.43 | 36.08 | 31.38 | 31.12 | 32.31 | 31.38 |
| 100 | 38.82 | 78.30 | 39.79 | 36.61 | 43.37 | 42.94 | 37.08 | 74.64 | 49.17 | 37.08 | 36.75 | 39.36 | 37.08 |

Table S5b: Predicted AMDR (mm) for the three machine learning models at standard return periods. Values represent the model's prediction at each return period's corresponding exceedance probability (P = 1/T).

| T (yr) | XGBR | RF | SVM |
| --- | --- | --- | --- |
| 2 | 6.00 | 6.00 | 6.00 |
| 5 | 11.00 | 10.00 | 10.00 |
| 10 | 18.00 | 17.00 | 18.00 |
| 25 | 36.00 | 31.00 | 28.00 |
| 50 | 45.00 | 41.00 | 32.00 |
| 100 | 46.00 | 42.00 | 35.00 |

S6. Uncertainty Analysis — 90% Bootstrap Confidence Interval Widths and Ranks

Table S6: 90% Bootstrap Confidence Interval width (mm) and uncertainty rank for all sixteen models at the six standard return periods (T = 2, 5, 10, 25, 50, and 100 years). Rank 1 denotes the narrowest CI (lowest uncertainty).

| Model | T = 2 yr | | T = 5 yr | | T = 10 yr | | T = 25 yr | | T = 50 yr | | T = 100 yr | |
| --- | --- | --- | --- | --- | --- | --- | --- | --- | --- | --- | --- | --- |
|  | CI (mm) | Rank | CI (mm) | Rank | CI (mm) | Rank | CI (mm) | Rank | CI (mm) | Rank | CI (mm) | Rank |
| *Statistical Distribution Models* | | | | | | | | | | | | |
| GEVMLE | 1.90 | 4 | 4.21 | 6 | 8.11 | 14 | 15.65 | 12 | 36.41 | 12 | 69.94 | 12 |
| GEVLM | 2.01 | 12 | 4.05 | 5 | 7.27 | 10 | 14.74 | 11 | 48.93 | 14 | 136.82 | 15 |
| GLLM | 1.98 | 10 | 3.95 | 4 | 7.96 | 12 | 20.39 | 15 | 81.87 | 16 | 166.45 | 16 |
| GPMLE | 2.39 | 14 | 4.79 | 12 | 7.09 | 9 | 11.29 | 8 | 21.84 | 10 | 36.09 | 10 |
| GPPM | 2.40 | 15 | 4.85 | 13 | 7.81 | 11 | 13.21 | 10 | 30.45 | 11 | 58.88 | 11 |
| GPM | 2.53 | 16 | 4.89 | 14 | 8.01 | 13 | 12.15 | 9 | 18.48 | 9 | 23.70 | 9 |
| GLM | 2.25 | 13 | 4.41 | 7 | 6.71 | 8 | 9.33 | 7 | 13.08 | 6 | 16.06 | 6 |
| EMLE | 1.93 | 7 | 4.47 | 9 | 6.39 | 5 | 8.31 | 3 | 10.86 | 3 | 12.78 | 3 |
| SEMLE | 1.92 | 5 | 4.47 | 11 | 6.43 | 7 | 8.35 | 5 | 10.93 | 5 | 12.89 | 5 |
| ELM | 1.93 | 7 | 4.47 | 9 | 6.39 | 5 | 8.31 | 3 | 10.86 | 3 | 12.78 | 3 |
| EPM | 1.93 | 7 | 4.47 | 9 | 6.39 | 5 | 8.31 | 3 | 10.86 | 3 | 12.78 | 3 |
| LLLM | 1.95 | 9 | 5.26 | 15 | 10.53 | 15 | 19.97 | 14 | 45.16 | 13 | 84.61 | 13 |
| LLPM | 1.99 | 11 | 5.73 | 16 | 11.93 | 16 | 24.52 | 16 | 59.85 | 15 | 116.24 | 14 |
| *Machine Learning Models* | | | | | | | | | | | | |
| RF | 0.20 | 2.5 | 1.00 | 2 | 2.00 | 1.5 | 9.00 | 6 | 18.00 | 8 | 20.00 | 8 |
| XGBR | 0.20 | 2.5 | 1.50 | 3 | 6.00 | 3 | 17.00 | 13 | 17.50 | 7 | 17.50 | 7 |
| SVM | 0.20 | 1 | 0.60 | 1 | 2.00 | 1.5 | 6.00 | 1 | 8.00 | 1 | 9.00 | 1 |

Table S7. Goodness-of-fit evaluation of twenty-five candidate statistical distributions fitted using the Probability Moments (PM), L-Moments (LM), and Maximum Likelihood Estimation (MLE) methods based on the Anderson–Darling (AD) and Kolmogorov–Smirnov (KS) goodness-of-fit tests. Models were classified as Accepted when the AD statistic was less than the critical value of 0.746 and the KS statistic was less than the critical value of 0.143 (α = 0.05, n = 91). Distributions yielding undefined (NaN) values were classified as Estimation Failed because parameter estimation did not converge during the optimization process.

| Distribution | Method | Anderson-Darling (Test Statistic) | Kolmogorov-Smirnov (Test Statistic | Status |
| --- | --- | --- | --- | --- |
| Generalized Pareto | PM | 0.072 | 0.08 | Accepted (1) |
|  | LM | NaN | 0.077 | Estimation Failed |
|  | MLE | 0.452 | 0.088 | Accepted (2) |
| Exponential | PM | 0.346 | 0.091 | Accepted (3) |
|  | LM | 0.346 | 0.091 | Accepted (4) |
|  | MLE | 0.346 | 0.091 | Accepted (5) |
| Log-Logistic | PM | 0.619 | 0.068 | Accepted (6) |
|  | LM | 0.659 | 0.068 | Accepted (7) |
|  | MLE | NaN | NaN | Estimation Failed |
| Gamma | PM | 0.085 | 0.111 | Accepted (8) |
|  | LM | 0.268 | 0.09 | Accepted (9) |
|  | MLE | 6.136 | 0.967 | Rejected |
| Log10-Normal | PM | 1.125 | 0.086 | Rejected |
|  | LM | 1.193 | 0.076 | Rejected |
|  | MLE | 1.125 | 0.086 | Rejected |
| Generalized Extreme Value | PM | 1.288 | 0.096 | Rejected |
|  | LM | 0.286 | 0.054 | Accepted (10) |
|  | MLE | 0.336 | 0.056 | Accepted (11) |
| Shifted Exponential | PM | 1.806 | 0.118 | Rejected |
|  | LM | NaN | 0.089 | Estimation Failed |
|  | MLE | 0.346 | 0.091 | Accepted (12) |
| Gumbel | PM | 3.438 | 0.157 | Rejected |
|  | LM | 2.129 | 0.116 | Rejected |
|  | MLE | 1.415 | 0.081 | Rejected |
| Logistic | PM | 5.557 | 0.183 | Rejected |
|  | LM | 5.161 | 0.159 | Rejected |
|  | MLE | 2.934 | 0.147 | Rejected |
| Generalized Logistic | PM | NaN | NaN | Estimation Failed |
|  | LM | 0.275 | 0.053 | Accepted (13) |
|  | MLE | NaN | NaN | Estimation Failed |
